# Very Low Prevalence of Validated *kelch13* Mutations and Absence of *Histidine-Rich Protein* 2/3 Double Gene Deletions in South African Malaria-Eliminating Districts (2022–2024)

**DOI:** 10.4269/ajtmh.25-0204

**Published:** 2025-10-23

**Authors:** Jaishree Raman, Maxwell Mabona, Qedusizi Nyawo, Brighton Mangena, Gerdalize Kok, Lihle Mathaba, Gillian Malatje, Sonja B. Lauterbach, Takalani I. Makhanthisa, Hazel Gwarinda, Blaženka D. Letinić, Arinao Ndadza, Eric Raswiswi, Mbavhalelo Shandukani, Ednah Baloyi, Ziyanda Fekema, Arshad Ismail, Jonathan Featherston, Bryan Greenhouse, Jennifer L. Smith, Andres Aranda-Diaz

**Affiliations:** ^1^ Centre for Emerging Zoonotic and Parasitic Diseases, National Institute for Communicable Diseases, Johannesburg, South Africa;; ^2^ Wits Research Institute for Malaria, University of Witwatersrand, Johannesburg, South Africa;; ^3^ Institute for Sustainable Malaria Control, University of Pretoria, Pretoria, South Africa;; ^4^ KwaZulu-Natal Provincial Malaria Control Program, Jozini, South Africa;; ^5^ Elimination Eight, Windhoek, Namibia;; ^6^ Mpumalanga Provincial Malaria Control Program, Mbombela, South Africa;; ^7^ Centre for Vaccines and Immunology, National Institute for Communicable Diseases, Johannesburg, South Africa;; ^8^ National Department of Health, Pretoria, South Africa;; ^9^ Sequencing Core Facility, National Institute for Communicable Diseases, Johannesburg, South Africa;; ^10^ Department of Biochemistry and Microbiology, Faculty of Science, Engineering and Agriculture, University of Venda, Thohoyandou, South Africa;; ^11^ EPPIcenter Research Program, Department of Medicine, University of California San Francisco, San Francisco, California, USA;; ^12^ Malaria Elimination Initiative, University of California San Francisco, San Francisco, California, USA;; ^13^ Department of Epidemiology and Biostatistics, University of California San Francisco, San Francisco, California, USA;; ^14^ ISGlobal, Barcelona, Spain

## Abstract

South Africa is one of 25 countries identified by the WHO as having the potential to eliminate malaria in the near future. In response to the emerging threat of antimalarial resistance, South Africa has enhanced its surveillance programs to enable the mapping of the prevalence of resistance markers at the facility level. A total of 4,471 samples, collected between January 2022 and August 2024 from healthcare facilities and during active surveillance in malaria-eliminating districts in KwaZulu-Natal and Mpumalanga Provinces, were assessed for drug (mutations in the *kelch13*, *chloroquine resistance transporter* [*crt*], *multidrug resistance 1* [*mdr1*], *dihydrofolate reductase* [*dhfr*], and *dihydropterate synthase* [*dhps*] genes) and diagnostic (*histidine-rich protein 2/3* [*hrp2/3*] gene deletions) resistance markers using polymerase chain reaction testing and sequencing (Sanger or targeted amplicon) protocols. Validated markers of artemisinin partial resistance were rare, with the P574L mutation detected as a minor allele in two samples and the P553L mutation present in one sample. Of the 60 additional nonsynonymous mutations detected, the A578S (30/999) and the I494V (13/951) mutations were most prevalent. Almost all parasites assessed carried the *crtK76* (99.8%) and *mdr1N*86 (99.0%) alleles, suggesting susceptibility to chloroquine. The *dhfr* triple (99.9%) and *dhps* double (98.2%) mutations associated with pyrimethamine and sulfadoxine resistance, respectively, were close to fixation. No single or double *hrp2/3* gene deletions were detected. These findings suggest that the recommended treatments and diagnostics in South Africa are effective. However, the strong selection for antimalarial drug resistance markers across southern Africa, combined with high regional interconnectedness, emphasizes the need for sustained malaria molecular surveillance to support South Africa and the southern Africa region in achieving their elimination goals.

## INTRODUCTION

The WHO acknowledged the sustained progress made by South Africa in reducing its malaria burden, citing it as a key contributor to the E2025 initiative.^1^ This initiative prioritized 25 malaria-endemic countries, including South Africa, to receive additional support and technical guidance in their efforts to stop indigenous malaria transmission within their national boundaries by 2025. However, several programmatic, biological, and environmental factors, including the recent emergence and spread of diagnostic-^2^ and drug-resistant^3^ parasites, have prevented South Africa and other African countries from achieving this goal.

Artemisinin-based combination therapies (ACTs) are the WHO-recommended and preferred malaria treatment globally.^4^ Most infections in South Africa and Africa are treated with the ACT artemether–lumefantrine (Coartem®),^5^ which has been highly effective on the continent for more than 20 years.^6^ African *Plasmodium falciparum* (*P. falciparum*) parasites with mutations in the propeller domain of the *kelch13* gene, along with confirmed delayed parasite clearance caused by the artemisinin partial resistance phenotype, are now established in several countries across East Africa, Central Africa, and the Horn of Africa.^7^ Although delayed parasite clearance alone may have limited clinical relevance, because of the action of the partner drug, increased drug pressure heightens the risk of the emergence of partner drug resistance. Once partner drug resistance develops, treatment failures become likely, as seen in the Greater Mekong Region.^8^

Given the devastating impact that ACT-resistant parasites would have on the malaria-vulnerable African population, the WHO released an Africa-specific response strategy.^6^ Routine surveillance for drug susceptibility using therapeutic efficacy studies and monitoring the prevalence of molecular markers of resistance are core activities of this strategy. The need for surveillance has become more urgent since parasites carrying *kelch13* mutations and deletions in the *histidine-rich protein* 2/3 (*hrp2/3*) coding genes have been confirmed in the Horn of Africa.^9^ These deletions enable parasites to evade detection by widely used HRP2-based rapid diagnostic tests (RDTs),^10^^,^^11^ thereby preventing malaria diagnosis and treatment and facilitating the rapid spread of diagnostic- and drug-resistant parasites.

South Africa established a genomic surveillance program^12^ after a drug-resistant malaria epidemic that occurred during the 1999/2000 malaria season.^13^ The findings directly led to artemether–lumefantrine replacing sulfadoxine–pyrimethamine (SP) as the first-line treatment.^14^ This surveillance program was further strengthened in 2018 when the linkage of drug resistance profiles with individual patient data, as well as the mapping of resistance data at the healthcare facility level in malaria-eliminating districts in KwaZulu-Natal and Mpumalanga Provinces (Figure 1), was facilitated to support malaria-elimination activities.^15^ These eliminating districts were targeted for enhanced surveillance because they are at increased risk for the emergence of resistance, given their low, unstable transmission intensities.^7^ Surveillance for *hrp2/3* gene deletions was added to the program in 2022.

**Figure 1.**
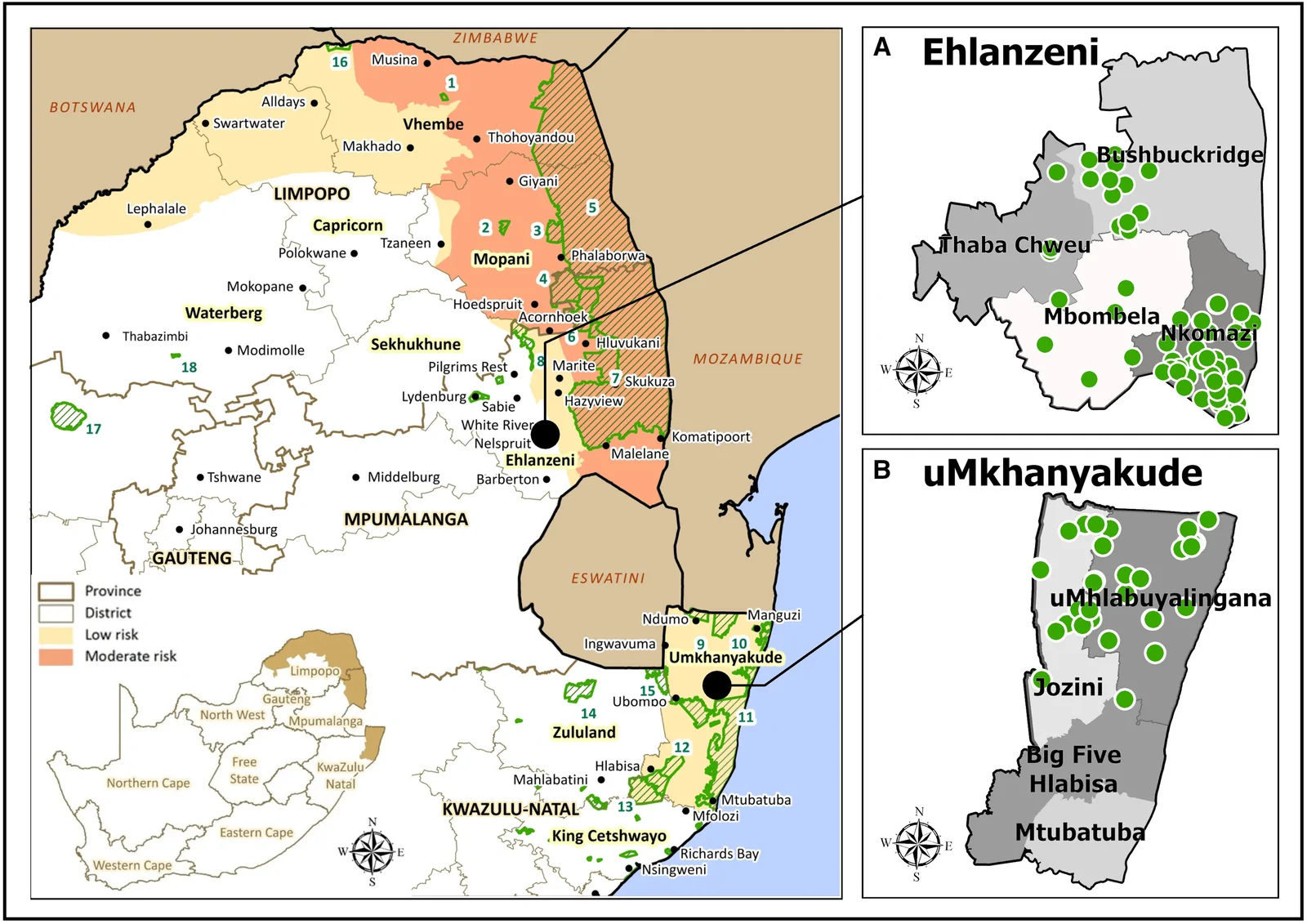
Malaria transmission areas in South Africa, with the healthcare facilities (highlighted in green) sampled from the (**A**) Ehlanzeni and (**B**) uMkhanyakude District Municipalities. ArcGIS Pro (Esri, Redlands, CA) was used to map the two districts where samples were collected, including healthcare facility locations. Shapefiles were sourced from the open-source ArcGIS platform (pro.arcgis.com, 2023).

The present genomic surveillance study, nested within the regional Genomics for Malaria in the Elimination 8 initiative, is aimed at generating near-real-time data on the prevalence and distribution of molecular markers of antimalarial drug and diagnostic resistance in South Africa, thereby informing case management responses. Findings on the prevalence of resistance markers in the malaria-eliminating districts of KwaZulu-Natal and Mpumalanga Provinces during the 2022/2023 and 2023/2024 malaria seasons, generated using published polymerase chain reaction (PCR) and next-generation sequencing (NGS) protocols, are presented in the current paper. The NGS workflow was included to gain deeper insights into the genomic epidemiology of malaria in these eliminating districts, as well as to assess its feasibility. Findings from the present study will inform diagnostic and treatment policies in these districts, thereby supporting malaria elimination efforts in South Africa.

## MATERIALS AND METHODS

### Country setting.

At the southernmost limit of malaria transmission in Africa, malaria in South Africa is restricted to the eastern low-altitude tropical border regions of three provinces, including Limpopo, Mpumalanga, and KwaZulu-Natal, which border Botswana, Mozambique, and Zimbabwe (Figure 1). Sustained implementation of effective vector control (primarily indoor residual spraying) and case management interventions, supported by rigorous surveillance, have significantly reduced malaria transmission, allowing the country to transition to an elimination agenda in 2012.^13^ Approximately 14.4% of the population resides in areas at risk for malaria transmission (South African Development Community, unpublished data), where transmission risk ranges from very low to moderate, depending on the locality.^16^ Most cases occur during the hot and humid wet months from September to March, with more than 90% of confirmed cases caused by *P. falciparum* infections^17^ and more than 80% of the cases in Mpumalanga and KwaZulu-Natal Provinces classified as imported on the basis of travel histories.^13^ The dominant vector is *Anopheles arabiensis*,^18^ whereas the secondary vector, *Anopheles merus*, contributes to transmission in certain areas.^19^

All individuals in malaria-endemic areas with a fever or a recent history of fever are tested for malaria either via HRP2-based falciparum-specific RDTs at primary healthcare facilities and during active surveillance in the communities or via microscopy at the hospital level.^20^ After a *P. falciparum* infection is confirmed, patients are either treated with the ACT artemether–lumefantrine for uncomplicated malaria or intravenous artesunate for severe malaria.^17^ In selected areas where elimination is targeted, a single low dose of the transmission-blocking antimalarial primaquine is co-administered with the first dose of artemether–lumefantrine to all eligible and consenting individuals.^17^

Malaria is a Category 1 notifiable condition in South Africa, requiring all cases to be reported within 24 hours of diagnosis through the Notifiable Medical Conditions and District Health Information System 2 (DHIS2) platforms. In addition, basic patient demographics and travel information are also captured on these platforms. In eliminating districts, cases should be investigated within 48 hours, with dried blood spots (DBSs) collected from patients, when possible, on days 3 and 28 to monitor treatment efficacy.

### Study sites and sample collection.

A total of 127 healthcare facilities, including 32 in the eliminating districts of the uMkhanyakude District Municipality, KwaZulu-Natal Province, and Ehlanzeni District Municipality and 95 in Mpumalanga Province, participated in ongoing data collection for surveillance studies during the 2022/2023 and 2023/2024 seasons. Filter paper (Whatman Filter No 1 [Cytiva, Marlborough, MA] or TFN blood cards [Ahlstrom_Munksjo, Borenstein, Germany]) finger-prick DBSs from all RDT-verified (First Response, Premier Technologies, Sarigam, India) malaria-positive individuals were collected by healthcare professionals based at these facilities, as well as malaria program staff, during active case detection activities in the community.

Routinely collected patient demographic and travel history data were linked to malaria-positive RDTs and associated DBSs using a unique barcode. Samples (including both malaria-positive RDTs with their associated DBSs and all malaria-negative RDTs) were shipped weekly from Mpumalanga Province and monthly from KwaZulu-Natal Province to the central malaria molecular laboratory at the National Institute for Communicable Diseases in Johannesburg, South Africa, for downstream genomic analysis.

### Molecular analysis.

At the central laboratory, all malaria-positive samples were entered into the genomic workflow, while 20% of the malaria RDT-negative samples received from each healthcare facility were randomly selected by the laboratory staff for quality assurance and *hrp2/3* gene deletion assessments. The random selection was not informed by a specific randomization method or software.

Parasite DNA extracted from the collected RDTs or DBSs using either the modified Chelex method^21^ or the Qiagen DNA mini extraction kit (Qiagen, Hilden, Germany) was subjected to a multiplex *Plasmodium* species PCR^22^ assay to confirm a malaria infection and identify infecting species. Additionally, a subsequent *P*. *falciparum varATS* quantitative PCR (qPCR)^23^ assay was used to confirm the presence of and quantify *P*. *falciparum* parasitemia. All PCR- and qPCR-confirmed samples were added to the drug resistance workflow.

Polymorphisms in the *P*. *falciparum chloroquine resistance transporter* (*crt*) 72–76 amino acid haplotype and the *multidrug resistance 1* (*mdr1*) 86 amino acid were assessed using previously described qPCR^24^ and restriction fragment length polymorphism^25^ assays, respectively. Polymorphisms in the propeller domain of the *kelch13* gene were assessed using nested PCR, as described in Talundzic et al.,^26^ followed by Sanger sequencing (inqaba biotec, Pretoria, South Africa). The resulting *kelch13* sequences were aligned against a reference *P. falciparum kelch13* gene (XM_001350122.1) using a BLAST (NIH, Bethesda, MD) search and the ClustalW multiple-sequence tool (EMBL, Cambridge) to detect polymorphisms after codon 400 in the propeller domain of the *kelch13* gene, which is the genetic region that contains mutations associated with delayed parasite clearance.^27^ The *crt*72–76 haplotype qPCR and *mdr1*86 nested PCR assays were run in duplicate, with a third assay performed on any discordant results. Codons were classified as either pure wildtype, pure mutant, or mixed (both mutant and wildtype genotypes present in an individual sample).

A randomly selected subset of samples from both transmission seasons was also genotyped using the Multiplex Amplicons for Drug, Diagnostic, Diversity, and Differentiation Haplotypes Using High-Throughput Targeted Resequencing (MAD^4^HatTeR, University of California San Francisco, San Francisco, CA) amplicon deep sequencing workflow.^28^ Briefly, primer pools D1.1, R1.1 or R1.2, and R2.1 were used to amplify 241 targets across the parasite genome, and the resulting libraries were sequenced on an Illumina NextSeq 2000 instrument (Illumina, San Diego, CA) using 150 base pair paired-end reads. The MAD^4^HatTeR bioinformatic pipeline was used to infer alleles across all targets.^28^ Pool R1.2 is a subset of primers in R1.1, and only single nucleotide polymorphisms (SNPs) present in pool R1.2 are reported. In addition to the genes genotyped via the traditional PCR assays indicated above, SNPs in the following genes were assessed: *dihydrofolate reductase* (*dhfr), dihydropterate synthase* (*dhps*), *mdr2*, *coronin*, *apicoplast ribosomal protein S10*, *putative phosphoinositide-binding*, *ferredodin*, *exonuclease*, and PF3D7_1322700. Target sequences in the *lactose dehydrogenase* gene of the *Plasmodium vivax*, *Plasmodium ovale* (*P*. *ovale*), and *Plasmodium malariae* (*P*. *malariae*) genomes were used to identify these species and differentiate between *P*. *ovale wallikeri* and *P*. *ovale curtisi*. Microhaplotypes from highly heterozygous targets were used to estimate the complexity of infection (COI). Variations to the original protocol and bioinformatic pipeline are described in Eloff et al.^29^

A random selection of RDT and PCR falciparum-positive samples, as well as all RDT malaria-negative but PCR falciparum-positive samples with a parasite concentration of ≥50 parasites/*µ*L of blood, were assessed for *hrp2/3* gene deletions using previously published qPCR assays.^30^^,^^31^

### Data management.

Malaria case data were extracted from the DHIS2 at regular intervals, covering the period from January 2022 to August 2024. The extracted data included routinely collected case information, such as patient demographic characteristics, travel history, and case classification (imported, local, and locally imported). After data cleaning and deduplication, individual patient demographic data and their associated antimalarial drug resistance profiles were linked using unique barcodes. When possible, samples with missing, incomplete, or incorrectly captured barcodes were linked using a probabilistic fuzzy matching technique (R packages Fuzzyjoin v0.1.6 and Stringdist v0.9.12; R Foundation, Vienna, Austria). The data were then categorized to distinguish between the 2022–2023 season (January 2022 to April 2023) and the 2023–2024 season (May 2023 to August 2024).

## STATISTICAL ANALYSES

R Studio (using R version 4.3.2 [R Foundation]) was used to clean, manage, and analyze linked genomic and patient demographic data. Basic descriptive statistical analysis was conducted using summary statistics and measures of central tendency. The Pearson χ^2^ test was used to identify statistically significant differences (*P*-value <0.05) between and within seasons and provinces.

ArcGIS Pro (Esri, Redlands, CA) was used to map the two districts where samples were collected, including the locations of healthcare facilities. Shapefiles were sourced from the open-source ArcGIS platform (pro.arcgis.com, 2023).

Drug resistance prevalence was estimated as the proportion of samples carrying a marker or mutation out of the total number of positive samples with a valid genotype in the marker of interest. The prevalence of mixed genotypes was also calculated for every combination of genotypes observed in the same sample. To evaluate the association between patient demographic factors and genotypes of interest, a multivariate logistic regression analysis was conducted using a generalized linear model with a binomial distribution. The presence of a genotype was used as the binary outcome, and province, season, sex, age, and case classification were included as independent variables. This analysis was only performed for genotypes *dhps*K540, *mdr1*Y184F, and *mdr2*I492V and the demographic categories of age, sex, and case classification with sufficient samples for model convergence.

The COI and effective COI (eCOI; which accounts for within-sample relatedness) for each sample and population allele frequencies were estimated using the R package MOIRE v3.4.0 (R Foundation), which implements a Markov chain Monte Carlo-based approach to jointly infer sample COI and within-host relatedness, as well as population allele frequencies, using polyallelic genomic data. By modeling genetic data and accounting for experimental errors, MOIRE provides probabilistic estimates. The mean COI and the proportion of polyclonal infections within each population, along with their 95% CIs, were derived from the posterior distributions in MOIRE’s output. To assess associations between COI and eCOI and demographic groups, multivariate analysis was conducted using a generalized linear model with a Poisson distribution. The COI-1 or eCOI-1 was used as the outcome, and province/season, age group, parasitemia, sample sequencing depth, and case classification were used as independent variables. Differences in the percentage of polyclonal samples were also assessed using the same approach with a binomial distribution, where samples were classified as polyclonal if they had an individual probability of polyclonality >0.05.

## RESULTS

### Samples received.

Over the study period, 49,122 RDT-positive and -negative samples were received by the laboratory from 127 healthcare facilities, with 23,415 and 25,707 samples submitted during the 2022/2023 and 2023/2024 seasons, respectively (Supplemental Figure 1). Most samples in each season (19,322 and 19,703) came from the Ehlanzeni District Municipality in Mpumalanga Province, with at least 90% of RDT-positive samples from both provinces classified as imported (KwaZulu-Natal Province: 93.7%, 477/509; Mpumalanga Province: 89.8%, 2,023/2,253). Of the 4,569 RDT-positive and 10,470 RDT-negative samples entered into the analytical workflow, 81.8% and 7.0% were confirmed as falciparum-positive via qPCR in KwaZulu-Natal and Mpumalanga Provinces, respectively (Table 1 and Supplemental Figure 1). During the 2023/2024 season, a higher proportion of RDT-negative samples were found to be malaria-positive via PCR in both provinces (Table 1). Most (94.7%, 640/676) of these RDT-negative but PCR-positive samples had concentrations of <50 parasites/*µ*L of blood (Supplemental Figure 2).

**Table 1:** Number of rapid diagnostic tests and dried blood spots collected from KwaZulu-Natal and Mpumalanga Provinces, South Africa, during the 2022/2023 and 2023/2024 malaria seasons, as well as the number confirmed as falciparum-positive via quantitaive polymerase chain reaction testing

Province	Season	Number of Samples Analyzed		Number of Falciparum-Positive Samples by qPCR (%)	
		Malaria RDT-Positive *N* = 4,569	Malaria RDT-Negative *N* = 10,470	Malaria RDT-Positive *N* = 3,738 (81.8%)	Malaria RDT-Negative *N* = 733 (7.0%)
KwaZulu-Natal	2022/2023	692	2,593	503 (72.7)	70 (2.7)
	2023/2024	548	770	445 (81.2)	124 (16.1)
Total		1,240	3,363	948 (76.5)	194 (5.8)
Mpumalanga	2022/2023	1,594	3,801	1,297 (81.4)	56 (1.5)
	2023/2024	1,735	3,306	1,493 (86.1)	483 (14.6)
Total		3,329	7,107	2,790 (83.8)	539 (7.6)
Grand total		15,039		4,471 (29.7%)	

qPCR = quantitative polymerase chain reaction; RDT = rapid diagnostic test.

Standard PCR assays only detected *P*. *falciparum* and *P*. *ovale* coinfections in less than 0.1% of the 4,471 *P*. *falciparum* qPCR-positive samples. In contrast, 51 of 2,405 samples processed with the MAD^4^HatTeR protocol corresponded to *P*. *falciparum* coinfections with *P*. *malariae*, *P*. *ovale curtisi*, and *P*. *ovale wallikeri* (Supplemental Table 1). Most coinfections were detected in KwaZulu-Natal Province, where they accounted for 9% of the samples, compared with 2% from Mpumalanga Province.

### Case characteristic.

With the exception of the collection method, sociodemographic data for confirmed falciparum cases were often incomplete (Table 2). Most of the falciparum-confirmed samples received by the central laboratory over the two malaria seasons were from males (58.9%) aged 10 years and above (Table 2). The sample collection method differed significantly between the two provinces: 72% of cases in Mpumalanga Province were detected through passive surveillance, whereas 77% of cases in KwaZulu-Natal Province were collected through active case detection (*P* <0.001; Table 2). Both provinces reported high numbers of imported cases, with imported cases being slightly more common in KwaZulu-Natal Province (94%) compared with Mpumalanga Province (90%; *P* = 0.014). Although citizenship data were not frequently captured, when they were, most participants were Mozambican (70.1%), followed by South African (29.4%, Table 2). In KwaZulu-Natal Province, the proportion of imported and actively detected cases increased significantly between the 2022/23 and 2023/24 seasons, whereas Mpumalanga Province exhibited a decreasing trend.

**Table 2:** Sociodemographic characteristics of the confirmed falciparum-positive study population in KwaZulu-Natal and Mpumalanga Provinces, South Africa, during the 2022/2023 and 2023/2024 malaria seasons

Sociodemographic Characteristics	Province						Overall		
	KZN			MPN					
	2022/2023 (*N* = 573)	2023/2024 (*N* = 569)	*P*-Value	2022/2023 (*N* = 1,350)	2023/2024 (*N* = 1,979)	*P*-Value	KZN (*N* = 1,142)	MPN (*N* = 3,329)	*P*-Value
Sex									
Female	200 (47%)	199 (38%)	**0.011**	558 (44%)	440 (37%)	**<0.001**	399 (42%)	998 (41%)	0.4
Male	229 (53%)	319 (62%)		715 (56%)	741 (63%)		548 (58%)	1,456 (59%)	
Unknown*	144	51		77	798		195	875	
Age category (years)									
0 to <5	17 (3.1%)	25 (6.0%)	0.3	139 (11%)	120 (7.7%)	**<0.001**	42 (4.4%)	259 (9.3%)	**<0.001**
5 to <10	63 (12%)	44 (11%)		156 (13%)	266 (17%)		107 (11%)	422 (15%)	
10 to <20	218 (40%)	163 (39%)		386 (32%)	491 (31%)		381 (40%)	877 (31%)	
20 to <30	121 (22%)	92 (22%)		266 (22%)	270 (17%)		213 (22%)	536 (19%)	
30 to <40	114 (21%)	78 (19%)		206 (17%)	321 (21%)		192 (20%)	527 (19%)	
≥40	13 (2.4%)	13 (3.1%)		72 (5.9%)	96 (6.1%)		26 (2.7%)	168 (6.0%)	
Unknown*	27	154		125	415		181	540	
Case classification									
Imported	193 (89%)	284 (97%)	**<0.001**	946 (93%)	1,077 (87%)	**<0.001**	477 (94%)	2,023 (90%)	**0.014**
Local	24 (11%)	8 (2.7%)		68 (6.7%)	152 (12%)		32 (6.3%)	220 (9.8%)	
Locally imported	0 (0%)	0 (0%)		1 (<0.1%)	9 (0.7%)		0 (0%)	10 (0.4%)	
Unknown*	356	277		335	741		633	1,076	
Method of detection									
Active	412 (72%)	466 (82%)	**<0.001**	437 (32%)	506 (26%)	**<0.001**	878 (77%)	943 (28%)	**<0.001**
Passive	161 (28%)	103 (18%)		913 (68%)	1,465 (74%)		264 (23%)	2,378 (72%)	
Unknown*	–	–		0	8		0	8	
Citizenship									
Mozambique	167 (91%)	48 (98%)	0.4	10 (67%)	10 (67%)	>0.9	215 (93%)	20 (67%)	**<0.001**
South Africa	15 (8.2%)	1 (2.0%)		5 (33%)	4 (27%)		16 (6.9%)	9 (30%)	
Eswatini	0 (0%)	0 (0%)		0 (0%)	1 (6.7%)		0 (0%)	1 (3.3%)	
Malawi	1 (0.5%)	0 (0%)		0 (0%)	0 (0%)		1 (0.4%)	0 (0%)	
Unknown*	390	520		1,335	1,964		910	3,299	

KZN = KwaZulu-Natal Province; MPN = Mpumalanga Province.

* Data are missing in the malaria information systems (Notifiable Medical Conditions Surveillance System and District Health Information System 2), and they are not included in any of the epidemiological analyses.

*P*-values in bold indicate statistical significance.

### Molecular analysis.

Of the 4,471 qPCR falciparum-positive samples, 3,874 were assayed for *kelch13* polymorphisms via Sanger sequencing, with 2,700 (70%) yielding valid results; 2,178 were assayed for the *crt*72–76 haplotype via qPCR, with 1,526 (70%) yielding valid results; and 1,054 were assayed for *mdr1*N86Y via restriction length fragment polymorphisms, with 473 (45%) yielding valid results. A total of 2,405 confirmed falciparum samples were added to the MAD^4^HatTeR workflow, of which 1,791 (74.5%) yielded a genotype for at least 15 SNPs of interest. The median sequencing depth per sample was 363,984 (interquartile range [IQR]: 78,385–1,583,111), with a median per-target depth of 646 (IQR: 70–3,844). A median of 213 of 238 (IQR: 122–225) targets had more than 100 reads. The SNPs with the lowest genotyping rates were *crt*218 and *crt*220 (549 samples); PF3D7_1322700 236 (1,166 samples); and *mdr1*86 (1,220 samples). The demographic characteristics of the successfully sequenced samples were similar to those of the falciparum-confirmed samples (Supplemental Table 2).

### *Kelch13* mutations.

*Kelch13* sequence data were generated from 2,453 (57%) samples using either Sanger sequencing or MAD^4^HatTeR. All mutations detected using either technique are listed in Table 3 and Supplemental Tables 3 and 4. In instances in which samples were assessed using both workflows, all mutations detected were reported. Samples carrying any *kelch13* mutation (validated, candidate, unevaluated, or not associated) were rare, with 63 (2.65%) of the samples having nonsynonymous mutations in one of 12 amino acids over the study period (Table 3 and Supplemental Tables 3 and 4). Two validated markers^32^ of artemisinin partial resistance were observed in Mpumalanga Province during the 2023/2024 season. The P574L mutation was a minor allele (within-sample low-frequency reads) in two samples, and the P553L mutation was a unique allele in one sample. The most common *kelch13* mutations were the A578S and I494V mutations (Table 3). The A578S mutation was observed in both provinces and seasons in less than 6% of the samples, with allele frequencies below 1.5%, whereas the I494V mutation was detected in KwaZulu-Natal Province during both seasons and Mpumalanga Province during the 2022/2023 season in less than 4% of the samples, with allele frequencies below 1% (Table 3 and Supplemental Table 3). The prevalence of all detected *kelch13* mutations is presented in Supplemental Table 4.

**Table 3:** Prevalence of mutations in the *kelch13, chloroquine resistance transporter, multidrug resistance 1, dihydrofolate reductase*, and *dihydropterate synthase* genes in KwaZulu-Natal and Mpumalanga Provinces, South Africa, during the 2022/2023 and 2023/2024 malaria seasons

Gene	Codon	Province							
		KwaZulu-Natal				Mpumalanga			
		2022/2023		2023/2024		2022/2023		2023/2024	
		*n/N*	%	*n/N*	%	*n/N*	%	*n/N*	%
*kelch13*	Any nonsynonymous mutation*	9/258	3.49	18/279	6.45	24/391	6.14	28/856	3.27
	V494I	3/147	2.04	7/182	3.85	3/198	1.52	0/424	0.00
	P553L	0/69	0.00	0/151	0.00	0/171	0.00	1/371	0.27
	P574L	0/175	0.00	0/182	0.00	0/210	0.00	2/432	0.46
	A578S	5/175	2.86	8/182	4.40	11/210	5.24	6/432	1.39
*crt*	F48L	0/184	0	2/278	0.72	0/383	0	5/854	0.59
	CVMNK 72–76 CVIET	0/225	0	0/279	0	0/389	0	3/857	0.35
*mdr1*	N86Y	0/114	0	0/168	0	11/289	3.81	1/645	0.16
	N86F	0/114	0	0/168	0	12/289	4.15	0/645	0
	Y184F	92/145	63.45	148/230	64.35	252/359	70.19	560/824	67.96
*mdr2*	I492V	45/130	34.62	58/210	27.62	94/317	29.65	235/723	32.5
*dhfr*	N51I	144/144	100	243/243	100	331/334	99.1	780/781	99.87
	C59R	144/144	100	243/243	100	334/334	100	779/781	99.74
	S108N	159/159	100	263/263	100	370/370	100	839/839	100
*dhps*	S436A	5/165	3.03	1/263	0.38	2/380	0.52	8/847	0.94
	S436F	3/165	1.82	1/263	0.38	1/380	0.26	1/847	0.12
	A437G	164/165	99.39	258/263	98.1	372/380	97.89	830/847	97.99
	K540E	137/140	97.86	236/239	98.74	330/337	97.92	756/774	97.67
	A581G	0/232	0	1/279	0.36	1/379	0.26	5/847	0.59
	A613S	1/227	0.44	0/263	0	0/358	0	2/791	0.25
	A613V	0/227	0	0/263	0	0/358	0	2/791	0.25

*crt* = *chloroquine resistance transporter*; *dhfr* = *dihydrofolate reductase*; *dhps* = *dihydropterate synthase*; *mdr* = *multidrug resistance*.

* All nonsynonymous SNPs (validated, candidate, unevaluated, and not associated) detected in the *kelch13* gene via Sanger sequencing and Multiplex Amplicons for Drug, Diagnostic, Diversity, and Differentiation Haplotypes Using High-Throughput Targeted Resequencing (University of California San Francisco, San Francisco, CA).

Sanger sequencing and MAD^4^HatTeR produced comparable results for *kelch13* mutations. Among 1,257 samples with results from both methods, 96% of the results were fully concordant. The MAD^4^HatTeR approach led to the detection of a mixed genotype in 49 samples (3.9%), which had a unique genotype according to Sanger sequencing, most of which had within-sample allele frequencies below 50% for the discordant allele (Supplemental Figure 3). Only one sample exhibited a unique allele that differed between the two methods.

### Other drug resistance mutations.

Mutations in the *dfhr* and *dhps* genes were only assessed through the MAD^4^HatTeR pipeline. The prevalence of mutations in the *dfhr* and *dhps* genes, which are associated with resistance to pyrimethamine and sulfadoxine, respectively, was high in both provinces across both seasons (Tables 3 and 4 and Supplemental Table 3). All samples from KwaZulu-Natal Province carried the *dhfr* triple haplotype (*dhfr*N51I+*dhfr*C59R+*dhfr*S108N) associated with pyrimethamine resistance, whereas only 99% of the samples from Mpumalanga Province carried this haplotype.^33^ The *dhfrI*164L mutation, which is associated with very high levels of pyrimethamine resistance,^34^ was not detected in any of the samples analyzed over the study period.

**Table 4:** Proportion (%) of *dihydropterate synthase* and *dihydrofolate reductase* mutations as haplotypes in KwaZulu-Natal and Mpumalanga Provinces, South Africa, during the 2022/2023 and 2023/2024 malaria seasons

Gene	Mutation	Province							
		KwaZulu-Natal				Mpumalanga			
		2022/2023		2023/2024		2022/2023		2023/2024	
		*N*	%	*N*	%	*N*	%	*N*	%
*dhps* + *dhfr*	<5 Mutations	145	2.07	240	1.67	336	2.68	762	2.62
	Quintuple		91.03		94.58		92.26		89.76
	Sextuple		0		0		0.6		0.52
	Undetermined		6.9		3.75		4.46		7.09
*dhfr*	<3 Mutations	158	0	260	0	360	1.11	826	0.36
	Triple		100		100		98.89		99.64
	Undetermined		–		–		–		–
*dhps*	<2 Mutations	145	2.07	240	1.25	339	0.88	765	0.26
	Double		91.03		94.58		92.92		90.46
	Triple		0		0		0.59		0.65
	Undetermined		6.9		3.75		4.42		6.67

*dhfr* = *dihydrofolate reductase*; *dhps* = *dihydropterate synthase*.

The *dhps* double haplotype (*dhps*A437G+*dhps*K540E) associated with sulfadoxine resistance in southern Africa,^35^ was found in more than 90% of the samples assessed in both provinces (Tables 3 and 4 and Supplemental Table 3). Up to 7% of the samples had an undetermined haplotype due to the presence of mixed microhaplotypes in polyclonal infections. The number of samples with the quintuple mutation (*dhfr*N51I+*dhfr*C59R+*dhfr*S108N+*dhps*A437G+*dhps*K540E) linked with SP treatment failures in East and southern Africa^36^ mirrored the *dhps* number (Tables 3 and 4 and Supplemental Table 3), given the fixation of the *dhfr* triple haplotype. Less than 1% of the samples carried mutations at codons *dhps*436 or *dhps*581 (Tables 3 and 4 and Supplemental Table 3).

Because of low genetic diversity in *dhps* and *dhfr* haplotypes, the analysis of the association between mutations and demographic factors was limited to the *dhps*K540 wildtype genotype. This genotype was more frequently found in local cases than in imported cases, with an odds ratio (OR) of 3.58 (95% CI: 2.04–6.30; Supplemental Table 5). Conversely, participants aged 40 or older were less likely to carry the genotype than those aged 4 years or younger, with an OR of 0.32 (CI: 0.12–0.89). No association was found with sex or other age categories.

The *crt*K76 and *mdr*1N86 alleles associated with full susceptibility to chloroquine^37^ but reduced susceptibility to lumefantrine^38^^,^^39^ remained close to fixation over the two seasons in both provinces (Table 3 and Supplemental Tables 3 and 4). Concordance between the two workflows was high for the *crt*72-76 wildtype (CVMNK) and mutant (CVIET) haplotypes, with only two of 573 (0.35%) samples genotyped using both methods producing discordant results. Both samples were classified as a mixed genotype according to MAD^4^HatTeR and a pure wildtype genotype according to qPCR. There was 100% concordance among 55 samples genotyped for *mdr1*N86Y via PCR and MAD^4^HatTeR. The *mdr1*Y184F mutation was found in 67.5% of the samples (Table 3) and was less likely to be found in locally transmitted infections, with an OR of 0.62 (CI: 0.42–1.16; Supplemental Table 6), with no significant associations with age or sex. Finally, the *mdr2*I492V mutation was found in 31.3% of the samples (Table 3) and was more likely to be carried by participants aged 5 to 9 years, with an OR of 2.19 (CI: 1.05–4.56, Supplemental Table 5). No significant associations were found with other age groups, sex, or case classification.

### *Histidine-rich protein 2/3* deletions.

No single or double *hrp2* and *hrp3* gene deletion*s* were detected in the 603 RDT-negative but PCR-positive samples assessed.

### Within-host genetic diversity.

Overall, the mean COI was higher in KwaZulu-Natal Province (3.14; CI: 3.03–3.23) than in Mpumalanga Province (2.83; CI: 2.78–2.88). The eCOI was similar between the two provinces (KwaZulu-Natal Province: 2.04; CI: 2.02–2.05; Mpumalanga Province: 2.03; CI: 2.02–2.04). The proportion of polyclonal infections also did not differ significantly (KwaZulu-Natal Province: 71.4%; CI: 69.3–73.2%; Mpumalanga Province: 68.5%; CI: 67.4–70.0%). Furthermore, the COI and eCOI in KwaZulu-Natal Province were significantly higher in the 2022/2023 season than in the 2023/2024 season (Figure 2 and Supplemental Table 6), although the proportion of polyclonal infections was not significantly different. All three metrics were significantly lower in locally acquired cases compared with imported cases (Figure 2 and Supplemental Table 6). Multivariate analysis adjusting for sequencing read depth, parasitemia, and age confirmed these results (Supplemental Table 7). The analysis also revealed that samples from participants older than 40 years had lower COI and eCOI values compared with those from participants under 5 years of age (Supplemental Table 7).

**Figure 2.**
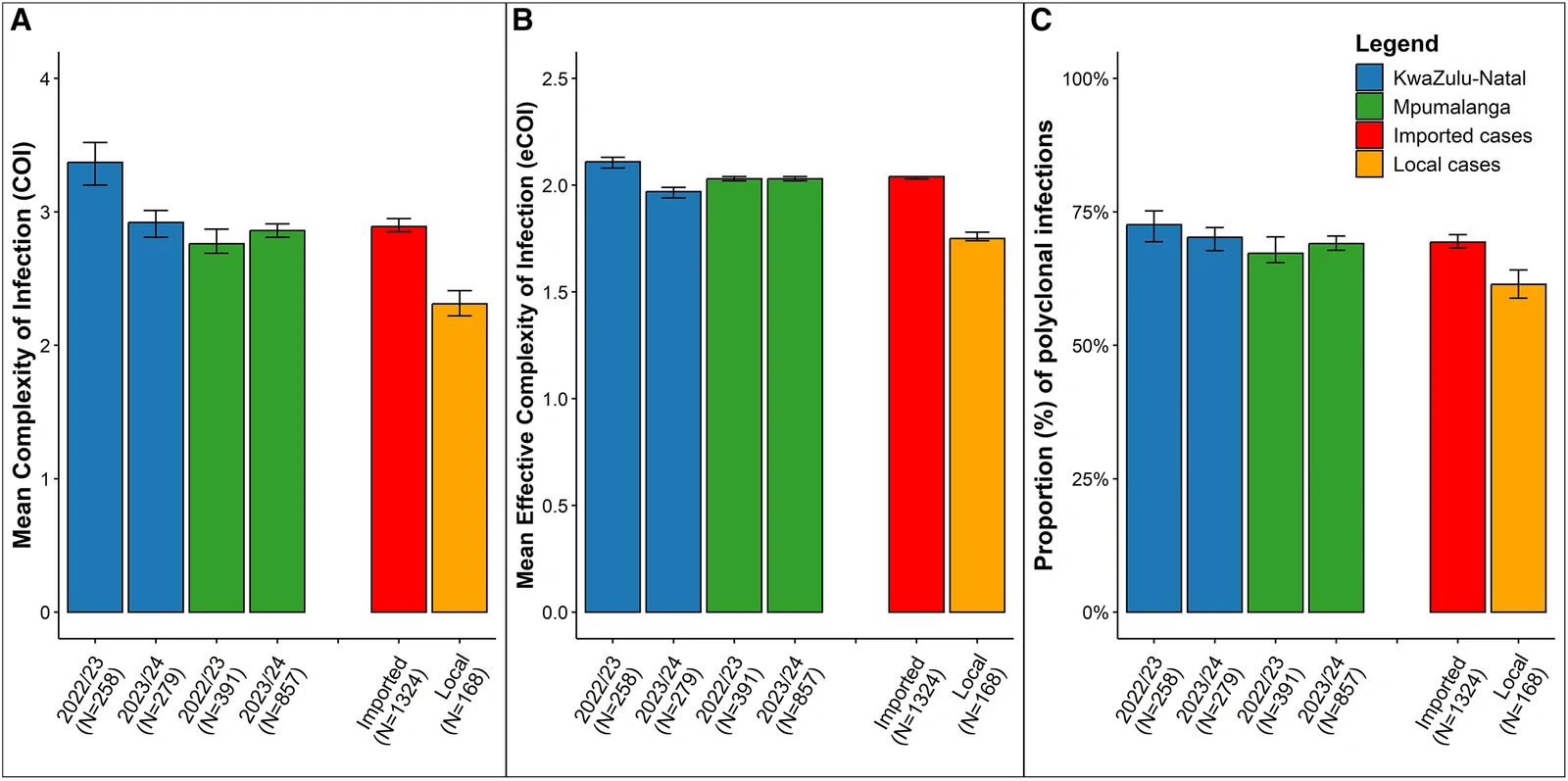
(**A**) Complexity of infection (COI), (**B**) effective COI, and (**C**) proportion of polyclonal infections in KwaZulu-Natal and Mpumalanga samples from the 2022/2023 and 2023/2024 malaria seasons, as well as in imported and local cases overall. Error bars represent 95% CIs.

## DISCUSSION

Despite more than 15 years of sustained artemether–lumefantrine use in South Africa,^40^ the results from the present study reveal a low prevalence of known markers of artemisinin partial resistance. These findings confirm the results of previous genomic surveillance^12^ and drug efficacy^41^ studies conducted in the country. Mutations in the *kelch13* gene previously detected in South Africa^42^^,^^43^ were not found in the present study, suggesting that resistant parasites either emerged de novo or were sporadically introduced through malaria importation, with limited or no selective advantage. This contrasts with the trend observed in other southern African countries, where *kelch13* mutations associated with artemisinin partial resistance have become established^29^^,^^44^^,^^45^ and spread rapidly.^32^^,^^46^

The most prevalent *kelch13* mutation detected in this study, A578S, is not associated with artemisinin partial resistance.^32^ The reason for its sustained selection requires further investigation. However, the detection of two validated markers of artemisinin partial resistance, *kelch13* P574L and P553L, in Mpumalanga Province during the 2023/2024 season is concerning. The prevalence of these and other novel mutations, as well as their impact on treatment efficacy, must be closely monitored. Surveillance efforts should be strengthened in areas where mutations have been detected. Therapeutic efficacy studies should be conducted in areas with high rates of treatment failures or increased prevalence of *kelch13* mutations, as recommended by the WHO.^6^ It is imperative that South Africa develops a response and migration plan to help keep its elimination ambitions on track.

Polymorphisms in the *P. falciparum mdr1* gene modulate susceptibility to several antimalarials, including lumefantrine and amodiaquine.^47^^,^^48^ In areas with prolonged artemether–lumefantrine use, selection for the *mdr1*N86 allele is observed,^12^^,^^39^ a trend observed in this study. Parasites with this wildtype allele display lower ex vivo susceptibility to lumefantrine compared with parasites that have the mutant *mdr1*86Y allele.^49^ Although a meta-analysis of clinical trial data revealed an association between the *mdr1*N86 allele and treatment failures,^39^ the change in lumefantrine susceptibility linked to the *mdr1*N86 allele is modest,^50^ potentially requiring additional mutations for treatment failure.

Although there have been no confirmed reports of treatment failure with artemether–lumefantrine in South Africa, data from two countries within the Southern African Development Community, Angola^51^ and the Democratic Republic of Congo,^52^ reveal reduced artemether-lumefantrine efficacy. This is particularly concerning for South Africa. Like in most African countries, artemether–lumefantrine is the first-line treatment for uncomplicated malaria,^6^ and currently, South Africa does not have a registered second-line ACT treatment available. South Africa must therefore intensify its efforts to protect the efficacy of artemether and lumefantrine. The safeguarding of these drugs has become more urgent because the next generation of antimalarials expected to enter the market in the next 2 years contains lumefantrine (ganaplacide plus lumefantrine and artemether–lumefantrine plus amodiaquine).

As seen in other African countries, the withdrawal of chloroquine as the preferred treatment for uncomplicated falciparum malaria resulted in the selection of the wildtype *P. falciparum crt*K76 genotype in South Africa. This wildtype allele is associated with increased susceptibility to chloroquine.^53^^,^^54^ The reemergence of chloroquine-susceptible parasites has sparked interest in reintroducing chloroquine for the treatment of falciparum malaria, particularly in areas where artemisinin partial resistance has been confirmed. The prevalence of chloroquine-resistant parasites in and around the country should inform this decision, as chloroquine resistance is likely to be rapidly selected in areas where resistant parasites are present.

Although SP has not been used in South Africa for malaria treatment or chemoprophylaxis since the late 2000s,^55^ mutations associated with resistance to sulfadoxine and pyrimethamine remain close to fixation.^12^ There are several potential drivers for the sustained selection of these mutations, including 1) sustained regional SP drug pressure through its use in chemoprevention activities in many neighboring countries, 2) cross-reaction with cotrimoxazole, an antifolate-sulfonamide combination used to treat opportunistic infections, particularly in patients with HIV/AIDS,^56^ and 3) the parasites circulating in South Africa carrying compensatory mutations that minimize the fitness costs of SP mutations. The association of the wildtype *dhps*K540 allele, which is linked to lower sulfadoxine resistance, with local transmission may reflect lower selective pressure in South Africa compared with Mozambique, where most imported cases originate and SP chemoprevention is practiced. The reason for the higher prevalence of the mutant *dhps*540E allele in children under 5 years of age remains unclear. It may be a consequence of higher exposure to antifolate–sulfonamide-based drugs, but further research is required.

The WHO has reported that parasites carrying the *hrp2* and *hrp3* gene deletions are rare in Africa and are currently localized in the Horn of Africa.^11^ The present study revealed no evidence of selection for parasites carrying single or both the deletions, despite falciparum-specific RDTs being used at the primary healthcare level since 2001.^57^ Although similar findings were reported in neighboring Mozambique,^58^ a recent study in KwaZulu-Natal with a small sample size^59^ revealed a high proportion of parasites with the *hrp2* and *hrp3* double deletion. Possible reasons for the discrepancy include the assessment of differing populations, issues with sample quality, and differences in assay sensitivity and specificity. An in-depth investigation is urgently required to elucidate the cause of these differences. The large sample sizes in the present study, as well as the one conducted in Mozambique,^58^ support the continued use of *hrp2*-based RDTs as the point-of-care diagnostic tool in South Africa. However, the prevalence of the *hrp2/3* gene deletions must be closely monitored to ensure that the recommended point-of-care diagnostic tool can accurately detect malaria infections.

Parasite importation from areas with higher transmission rates can shape the genetic characteristics of local parasite populations. Higher transmission generally leads to greater population-level and intra-host genetic diversity. Consistent with this, the imported infections in the present study, which account for 90% of cases, predominantly among males from Mozambique, had higher COI than locally transmitted infections. As a result, high levels of polyclonality were observed in the eliminating districts of KwaZulu-Natal and Mpumalanga Provinces, aligning with findings previously reported in neighboring Eswatini.^60^ Previous studies have revealed that antimalarial drug-resistant parasites have been spread across Africa by highly mobile African populations.^36^^,^^61^^,^^62^ This highlights the increased risk of drug-resistant parasites being introduced in KwaZulu-Natal and Mpumalanga Provinces through importation, as most cases notified in these provinces^63^^,^^64^ are imported. Although molecular markers of artemisinin partial resistance are currently rare in South Africa, they are rapidly becoming established in several southern African countries, including Namibia^29^ and Zambia,^44^^,^^45^ emphasizing the need for proactive routine genomic surveillance, particularly along known migration routes, to guide prompt mitigation responses. Although there is no evidence that *kelch13* mutations detected in Namibia^29^ and Zambia^44^ contributed to recent upsurges in these two countries,^63^ the increase in cases in South Africa does not appear to be driven by artemisinin or lumefantrine resistance.

As seen in earlier studies conducted in these two provinces,^64^^,^^65^ more males were affected by malaria than females, a trend observed in several other malaria-endemic countries.^66^^,^^67^ It is likely that work-related and social activities increase their exposure to malaria vectors. In addition, males are more likely to travel to malaria-endemic areas in search of employment. This highlights the need for malaria awareness campaigns and interventions specifically targeting this high-risk group, particularly in malaria-eliminating areas.

Although all genotyping protocols produced comparable results, the MAD^4^HatTeR NGS workflow resulted in the detection of more minor alleles and non-falciparum coinfecting species, which could be attributed to differences in sensitivity. The clinical relevance of these minor resistance alleles is currently unclear; therefore, further research is required to understand their public health value. The high prevalence of falciparum coinfections with *P. ovale*, particularly in KwaZulu-Natal Province, warrants further investigation. Although artemether–lumefantrine can clear blood-stage ovale parasites, the antimalarial is ineffective against the dormant liver-stage ovale parasite, resulting in relapsing malaria episodes. Further investigations are required to determine the true burden of non-falciparum *Plasmodium* in South Africa and its neighboring countries to guide case management responses.

The feasibility of implementing the MAD^4^HatTeR NGS workflow for routine malaria molecular surveillance, as well as the genetic epidemiological insights it provides, was confirmed in the present study. However, the critical shortage of skilled bioinformaticians with malaria genomics expertise in southern Africa, as well as the lack of secured funding, remains a significant obstacle to sustaining NGS workflows. Automated bioinformatics workflows aimed at improving accessibility are under development; however, in the interim, bioinformatics support from partners, primarily located in the Global North, remains necessary. Although traditional malaria molecular surveillance techniques, such as Sanger sequencing and qPCR, are individually cheaper than NGS and do not require advanced bioinformatics processing, they lack the resolution required to characterize polyclonal infections or are not scalable. Malaria programs must therefore select a genomic platform on the basis of information required for programmatic decision-making and resources available, while building the capacity to employ new technologies that generate data from multiple markers in a cost-efficient manner.

The present study has several limitations. First, resistance data were only generated from eliminating districts in Mpumalanga and KwaZulu-Natal Provinces. No resistance data were generated for Limpopo Province, which reports the highest number of locally acquired cases,^68^ because of several logistical and procedural challenges. Drug- and diagnostic-resistant parasites may be present there, contributing to the sustained malaria burden in that province. Acknowledging the value of malaria molecular surveillance for effective control, Limpopo Province recently committed to implementing genomic surveillance activities during the 2025/2026 malaria season.

Second, only validated resistance markers were assessed, potentially missing novel mutations associated with reduced drug efficacy. A random selection of samples should be subjected to whole-genome sequencing annually to proactively detect novel markers of resistance. Third, the absence of validated molecular markers for lumefantrine tolerance or resistance means that there is currently no molecular-based system for the early detection of reduced lumefantrine efficacy. Assessing parasite clearance on days 3 and 28 post-treatment is therefore essential for the prompt detection of potential treatment failures. Provincial programs must make every effort to collect these follow-up samples to ensure that decreases in drug efficacy can be detected and addressed in a timely manner. Fourth, the poor quality of blood samples collected, particularly from KwaZulu-Natal Province, limited the number of samples that could be analyzed using the NGS platform. Routine quality assessments of DBS collection should be conducted, with refresher training offered to facilities that submit suboptimal samples.

Lastly, epidemiological analyses were hindered by the absence of critical data variables in malaria information systems, such as travel history and sample barcodes. Possible reasons for this include an overburdened healthcare workforce performing multiple tasks and the lack of awareness among healthcare workers and malaria program staff of the value and importance of these data, particularly in malaria elimination settings. Raising awareness among healthcare workers and malaria program staff about the requirements for malaria elimination and the importance of collecting complete and accurate patient information must be prioritized, and regular training on data collection and reporting should be offered.

## CONCLUSION

The present study confirmed a low prevalence of *kelch13* mutations associated with artemisinin partial resistance and the absence of the *hrp2/3* double gene deletion associated with diagnostic resistance in eliminating districts of two South African malaria-endemic provinces. It is unclear whether the low prevalence of the *kelch13* mutation is due to limited selection pressure or the sporadic introduction by mobile migrant populations. However, the continued selection for the reduced lumefantrine susceptibility marker *mdr1*N86 in both provinces is concerning. Its entrenchment could potentially increase pressure on artemether to clear parasites, heightening the selection pressure for artemisinin partial resistance in South Africa. This threat, combined with the emergence and spread of *kelch13* mutations in several southern African countries, reinforces the need for routine malaria genomic surveillance in South Africa to guide timely responses to emerging resistance. The high prevalence of imported malaria in males must be addressed through targeted awareness campaigns and interventions.

## Supplemental Materials

10.4269/ajtmh.25-0204 Supplemental Materials
