# Supplementary material for: Very Low Prevalence of Validated kelch13 Mutations and Absence of Histidine-Rich Protein 2/3 Double Gene Deletions in South African Malaria-Eliminating Districts (2022–2024)
Source: Am J Trop Med Hyg. 2025 Oct 23;115(5 Suppl):53–64. doi: 10.4269/ajtmh.25-0204 (PMC13737031; doi:10.4269/ajtmh.25-0204)
Supplement: Supplementary file 1 [file tpmd250204.SD1.pdf]

Supplementary Tables

Table S1: Malaria species identified by standard PCR and the MAD[4]HatTeR protocols in KwaZulu-Natal and Mpumalanga provinces, during the 2022/2023 and 2023/2024 malaria seasons.

**PARASITE SPECIES**	**PROVINCE**						**OVERALL**		
	**KWAZULU-NATAL (KZN)**			**MPUMALANGA (MPN)**					
	**2022/2023**	**2023/2024**	***P*-Value**	**2022/2023**	**2023/2024**	***P*-Value**	**KZN**	**MPN**	***P*-Value**
**PCR**	*N* = 573	*N* = 569		*N* = 1,350	*N* = 1,979	0.4	*N* = 1,142	*N* = 3,329	>0.9
Pf[a]	573 (100%)	569 (100%)		1,349 (100%)	1,979 (100%)		1,142	3,328 (100%)	
Pf + Po[b]	0 (0%)	0 (0%)		1 (<0.1%)	0 (0%)		0 (0%)	1 (<0.1%)	
**MAD[4]HatTTeR**	*N* = 132	*N* = 162	0.7	*N* = 302	*N* = 710	0.8	*N* = 294	*N* = 1 012	<0.001
Pf[a]	119 (90%)	148 (91%)		297 (98%)	691 (97%)		267 (91%)	988 (98%)	
Pf + Pm[c]	9 (6.8%)	8 (4.9%)		4 (1.3%)	12 (1.7%)		17 (5.8%)	16 (1.6%)	
Pf + Pm + Poc[d]	0 (0%)	2 (1.2%)		0 (0%)	0 (0%)		2 (0.7%)	0 (0%)	
Pf + Pm + Pow[e]	0 (0%)	1 (0.6%)		0 (0%)	0 (0%)		1 (0.3%)	0 (0%)	
Pf + Poc[f]	2 (1.5%)	2 (1.2%)		0 (0%)	4 (0.6%)		4 (1.4%)	4 (0.4%)	
Pf + Poc + Pow[g]	0 (0%)	0 (0%)		0 (0%)	1 (0.1%)		0 (0%)	1 (<0.1%)	
Pf + Pow[h]	2 (1.5%)	1 (0.6%)		1 (0.3%)	2 (0.3%)		3 (1.0%)	3 (0.3%)	

[a] *Pf: Plasmodium falciparum*

[b] *Pf +Po: Plasmodium falciparum + Plasmodium ovale*

[c] *Pf +Pm: Plasmodium falciparum + Plasmodium malariae*

[d] *Pf + Pm + Po: Plasmodium falciparum + Plasmodium malariae + Plasmodium ovale*

[e] *Pf + Pm + Pow: Plasmodium falciparum + Plasmodium malariae + Plasmodium ovale wallikeri*

[f] *Pf + Poc: Plasmodium falciparum + Plasmodium ovale curtisi*

[g] *Pf + Poc + Pow: Plasmodium falciparum + Plasmodium ovale curtisi + Plasmodium ovale wallikeri*

[h] *Pf + Pow: Plasmodium falciparum + Plasmodium ovale wallikeri*

Table S2: Cross-tabulation of socio-demographic characteristics between confirmed falciparum samples and samples successfully sequenced using MAD^{4}HatTeR

Socio-demographic characteristics	Total number of qPCR positive samples	Number of successfully sequenced samples	*P*-Value
**Province**			
KwaZulu-Natal	701 (30%)	537 (30%)	>0.9
Mpumalanga	1,630 (70%)	1,248 (70%)	
Unknown	7	6	
**Malaria Season**			
2022/2023	916 (39%)	649 (36%)	**<0.001**
2023/2024	1,415 (61%)	1,136 (64%)	
Unknown	7	6	
**Gender**			
Female	640 (40%)	512 (41%)	**0.017**
Male	975 (60%)	730 (59%)	
Unknown	723	549	
**Age group**			
0 - <5	202 (8.8%)	163 (9.3%)	0.4
5 - <10	113 (4.9%)	92 (5.2%)	
10 - <20	294 (13%)	225 (13%)	
20 - <30	698 (30%)	521 (30%)	
30 - <40	484 (21%)	364 (21%)	
≥40	508 (22%)	392 (22%)	
Unknown	39	34	
**Case classification**			
Imported	1,678 (88%)	1,325 (88%)	0.8
Local	210 (11%)	170 (11%)	
Locally imported	13 (0.7%)	11 (0.7%)	
Unknown	437	285	

Table S3: Allele frequency for all genotypes assessed in falciparum samples from KwaZulu-Natal and Mpumalanga provinces, South Africa, during the 2022/2023 and 2023/2024 malaria seasons

Gene	Amino acid	Allele	PROVINCE			
			**KWAZULU-NATAL**		**MPUMALANGA**	
			**2022/2023**	**2023/2024**	**2022/2023**	**2023/2024**
dhfr	16/51/59	AIC	0.000237 (3.47e-08 - 0.00249)	0.00182 (4.76e-08 - 0.00694)	0.00412 (0.000877 - 0.0103)	0.00478 (0.00205 - 0.00873)
dhfr	16/51/59	AIR	0.997 (0.989 - 1)	0.998 (0.993 - 1)	0.989 (0.981 - 0.995)	0.988 (0.982 - 0.993)
dhfr	16/51/59	ANR	0.00289 (4.79e-05 - 0.011)	0	0.00666 (0.00232 - 0.0135)	0.00707 (0.00376 - 0.0114)
dhps	431/436/437	IAA	0.00712 (0.000772 - 0.0199)	0.000107 (1.58e-12 - 0.00119)	0.00129 (4.44e-05 - 0.00465)	0.00349 (0.00127 - 0.00703)
dhps	431/436/437	IAG	1.18e-11 (1.03e-12 - 7.13e-11)	0	0	4.23e-05 (2.94e-08 - 0.000407)
dhps	431/436/437	IFA	0.00266 (6.51e-05 - 0.0102)	0.000134 (3.05e-08 - 0.00127)	6.27e-05 (1.4e-12 - 0.000711)	0
dhps	431/436/437	IFG	5.31e-12 (1.02e-12 - 3.23e-11)	0	0	6.04e-05 (3.46e-08 - 0.000557)
dhps	431/436/437	ISA	0.0496 (0.0315 - 0.0751)	0.0215 (0.0107 - 0.0347)	0.0316 (0.0207 - 0.0448)	0.0463 (0.0357 - 0.0563)
dhps	431/436/437	ISG	0.941 (0.914 - 0.961)	0.978 (0.965 - 0.989)	0.967 (0.953 - 0.978)	0.95 (0.939 - 0.961)
dhps	431/436/437	ICA	0	0.000144 (3.36e-08 - 0.00145)	0	0
dhps	540/581	EA	0.946 (0.917 - 0.969)	0.967 (0.948 - 0.981)	0.952 (0.934 - 0.967)	0.942 (0.93 - 0.953)
dhps	540/581	KA	0.0543 (0.0313 - 0.0829)	0.0331 (0.0186 - 0.0522)	0.0468 (0.0323 - 0.0632)	0.0558 (0.0454 - 0.068)
dhps	540/581	EG	0	0	0.0014 (3.63e-05 - 0.00505)	0.00182 (0.000359 - 0.00408)
dhps	540/581	ET	0	0	0	7.84e-05 (3.25e-08 - 0.000703)
dhps	613	A	1 (1 - 1)	0	0	0.999 (0.997 - 1)
dhps	613	S	4.69e-08 (3.32e-08 - 5.52e-08)	0	0	0.000659 (2.01e-05 - 0.00245)
dhps	613	V	0	0	0	0.00059 (1.84e-05 - 0.00242)
k13	494	I	0.00224 (4.73e-05 - 0.00762)	0.00935 (0.00284 - 0.0188)	0.00347 (0.000554 - 0.00901)	0.00115 (0.000159 - 0.00325)
k13	494	V	0.998 (0.992 - 1)	0.991 (0.981 - 0.997)	0.997 (0.991 - 0.999)	0.999 (0.997 - 1)
k13	578	A	0.992 (0.983 - 0.998)	0.986 (0.976 - 0.994)	0.985 (0.975 - 0.993)	0.995 (0.991 - 0.997)
k13	578	S	0.00756 (0.00218 - 0.0168)	0.0137 (0.00576 - 0.0245)	0.0144 (0.00696 - 0.0244)	0.00534 (0.00257 - 0.00929)
k13	578	T	7.37e-05 (1.04e-12 - 0.000923)	0	0.000128 (3.3e-08 - 0.00113)	6.11e-05 (3.22e-08 - 0.000534)
mdr1	182/184/186	GFW	0.432 (0.365 - 0.497)	0.479 (0.421 - 0.532)	0.494 (0.451 - 0.537)	0.48 (0.449 - 0.51)
mdr1	182/184/186	GYW	0.568 (0.503 - 0.635)	0.521 (0.468 - 0.579)	0.506 (0.463 - 0.549)	0.52 (0.49 - 0.551)
mdr1	86	F	0	0	8.08e-12 (1.02e-12 - 2.73e-11)	0
mdr1	86	N	0	0	0.995 (0.988 - 0.999)	1 (1 - 1)
mdr1	86	Y	0	0	0.00516 (0.00115 - 0.0122)	1.61e-09 (0 - 2.9e-08)
mdr2	492	I	0.812 (0.762 - 0.862)	0.832 (0.791 - 0.871)	0.832 (0.8 - 0.862)	0.811 (0.787 - 0.833)
mdr2	492	V	0.188 (0.138 - 0.238)	0.168 (0.129 - 0.209)	0.168 (0.138 - 0.2)	0.189 (0.167 - 0.213)

Table S4: Prevalence of all *kelch13* mutation detection in KwaZulu-Natal and Mpumalanga provinces, South Africa, during the 2022/2023 and 2023/2024 malaria seasons

kelch13 mutation	Mutation classification*	PROVINCE							
		KWAZULU-NATAL				MPUMALANGA			
		2022/2023		2023/2024		2022/2023		2023/2024	
		n/N	**%**	***n/N***	**%**	***n/N***	**%**	***n/N***	**%**
V494I	Unevaluated[a]	3/147	2.04	7/182	3.85	3/198	1.52	0/424	0.00
P553L	Validated[b]	0/69	0.00	0/151	0.00	0/171	0.00	1/371	0.27
P574L	Validated[b]	0/175	0.00	0/182	0.00	0/210	0.00	2/432	0.46
A578S	No association[c]	5/175	2.86	8/182	4.40	11/210	5.24	6/432	1.39
A578T	Unevaluated[a]	1/175	0.57	0/182	0.00	0/210	0.00	1/432	0.23
C580R	Unevaluated[a]	0/175	0.00	1/182	0.55	1/210	0.48	0/432	0.00
V581A	Unevaluated[a]	0/175	0.00	2/182	1.10	0/210	0.00	0/432	0.00
L598S	Unevaluated[a]	0/174	0.00	1/178	0.56	1/203	0.49	0/416	0.00
N599D	Unevaluated[a]	0/174	0.00	0/178	0.00	0/203	0.00	1/416	0.24
E605V	Unevaluated[a]	0/174	0.00	1/178	0.56	0/203	0.00	0/416	0.00
K607E	Unevaluated[a]	0/174	0.00	1/178	0.56	1/203	0.49	0/416	0.00
K607R	Unevaluated[a]	0/174	0.00	0/178	0.00	3/203	1.48	2/416	0.48

*Classifications of *kelch13* mutations by the World Health Organization

[a]Unevaluated: a reported SNP but therapeutic impact not assessed by clinical or in vitro studies

[b]Validated: a SNP with evidence from clinical and in vitro studies of association with delayed parasite clearance

[c]No association: a SNP with no evidence from clinical and in vitro studies of association with delayed parasite clearance

Table S5: Multivariate logistic regression results for relevant demographics and drug resistance markers of interest

Characteristic	*dhps* K540			*mdr1* Y184F			*mdr2* I492V		
	n/N **(%)**	**OR[a] (95% CI[b])**	***P*** **-Value**	***n/N*** **(%)**	**OR[a] (95% CI[b])**	***P*** **-Value**	***n/N*** **(%)**	**OR[a] (95% CI[b])**	***P*** **-Value**
**Gender**	***N*** **= 950**			***N*** **= 1,004**			***N*** **= 885**		
Female	35/396 (8.83%)	Ref[c]		281/416 (67.54%)	Ref[c]		104/361 (28.80%)	Ref[c]	
Male	51/554 (9.20%)	1.09 (0.68 - 1.73)	0.7	406/588 (69.04%)	1.06 (0.80 - 1.39)	0.7	169/524 (32.25%)	1.19 (0.88 - 1.60)	0.3
**Age Group**									
0 -<5	10/105 (9.52%)	Ref[c]		79/111 (71.17%)	Ref[c]		23/96 (23.96%)	Ref[c]	
5 - <10	6/57 (10.52%)	0.68 (0.22-2.09)	0.5	49/64 (76.56%)	1.50 (0.73 - 3.09)	0.3	22/53 (41.51%)	2.19 (1.05 - 4.56)	**0.037**
10 - <20	22/140 (15.71%)	1.34 (0.59 - 3.04)	0.5	97/140 (69.28%)	1.01 (0.58 - 1.76)	>0.9	42/125 (33.60%)	1.58 (0.86 - 2.90)	0.14
20 - <30	22/278 (7.91%)	0.82 (0.37 - 1.82)	0.6	206/291 (70.79%)	0.98 (0.60 - 1.59)	>0.9	75/266 (28.20%)	1.20 (0.70 - 2.06)	0.5
30 - <40	19/191 (9.94%)	0.97 (0.43 - 2.21)	>0.9	130/197 (65.98%)	0.81 (0.49 - 1.35)	0.4	61/176 (34.66%)	1.69 (0.96 - 2.97)	0.069
≥40	7/179 (3.91%)	0.32 (0.12 - 0.89)	**0.029**	126/201 (62.68%)	0.70 (0.42 - 1.16)	0.2	50/169 (29.59%)	1.33 (0.75 - 2.37)	0.3
**Case classification**									
Imported	61/834 (7.31%)	Ref[c]		616/883 (69.76%)	Ref[c]		238/779 (30.55%)	Ref[c]	
Local	24/110 (21.81%)	3.58 (2.04 - 6.30)	**<0.001**	68/115 (59.13%)	0.62 (0.40 - 0.94)	**0.023**	35/100 (35.00%)	1.06 (0.67 - 1.69)	0.8
Locally imported	1/6 (16.66%)	3.54 (0.38 - 32.6)	0.3	3/6 (50.00%)	0.50 (0.10 - 2.53)	0.4	0/6 (0.00%)	0.00 (0.00 - inf)	>0.9

[a] *OR: Odds Ratio*

[b] *95% CI: 95% Confidence Interval*

[c] *Ref: Reference group*

Table S6: Within host genetic diversity in samples in KwaZulu-Natal and Mpumalanga provinces, South Africa, during the 2022/2023 and 2023/2024 malaria seasons.

Variable	PROVINCE				CASE CLASSIFICATION	
	KWAZULU-NATAL		MPUMALANGA		IMPORTED	LOCAL
	2022/2023	2023/2024	2022/2023	2023/2024	*N* = 1,322	*N* = 170
**COI[a]**	3.37 (3.20-3.52)	2.92 (2.81-3.01)	2.76 (2.69-2.87)	2.86 (2.81-2.91)	2.89 (2.85-2.95)	2.31 (2.22-2.41)
**eCOI[b]**	2.11 (2.08-2.13)	1.97 (1.94-1.99)	2.03 (2.02-2.04)	2.03 (2.02-2.04)	2.04 (2.03-2.04)	1.75 (1.74-1.78)
**% polyclonal**	72.59% (69.38-75.19%)	70.22% (67.74-72.05%)	67.25% (65.47-70.33%)	69.05% (67.79-70.48%)	69.31 % (68.23-70.73%)	61.42% (58.82-64.12%)

[a]COI: complexity of infection

[b]eCOI: effective COI

Mean (95% confidence interval)

Table S7: Multivariate logistic regression results for relevant demographics and COI, eCOI and polyclonality

Outcome	COI[a]		eCOI[b]		Probability polyclonal >0.05	
**Coefficients**	**Estimate**	***P*- Value**	**Estimate**	***P*- Value**	**Estimate**	***P*- Value**
**Intercept**	1,51E+00	**<0.001**	8,49E-01	**<0.001**	1,49E+00	**<0.001**
**KZN-2023/2024**	-5,80E-01	**<0.001**	-4,73E-01	**0.007**	-7,11E-01	0.083
**MPN-2022/2023**	-4,10E-01	**0,001**	-2,13E-01	0.197	-1,96E-01	0.623
**MPN-2023/2024**	-2,80E-01	**0,010**	-2,14E-01	0.1501	-2,85E-01	0.446
**log10(parasitemia)**	-1,77E-01	**<0.001**	-1,39E-01	**<0.001**	-1,03E-07	0.409
**Sequencing_depth**	2,71E-08	0.100	4,19E-08	0.035	2,34E-08	0.558
**Age:5-9**	1,92E-01	0.186	1,49E-01	0.402	NA	NA
**Age:10-19**	-2,13E-02	0.869	-1,33E-01	0.405	NA	NA
**Age:20-29**	-4,25E-03	0.970	-1,23E-01	0.379	NA	NA
**Age:30-39**	-7,49E-02	0,530	-2,47E-01	0.096	NA	NA
**Age:40+**	-2,43E-01	**0.045**	-4,46E-01	**0.004**	NA	NA
**Local**	-3,45E-01	**<0.001**	-2,80E-01	**0.029**	-4,89E-01	**0.033**
**Locally imported**	-1,30E-01	0.671	-3,32E-01	0,459	3,48E-02	0.966

[a]COI: complexity of infection

[b]eCOI: effective COI.

Supplementary Figures

Figure S1:

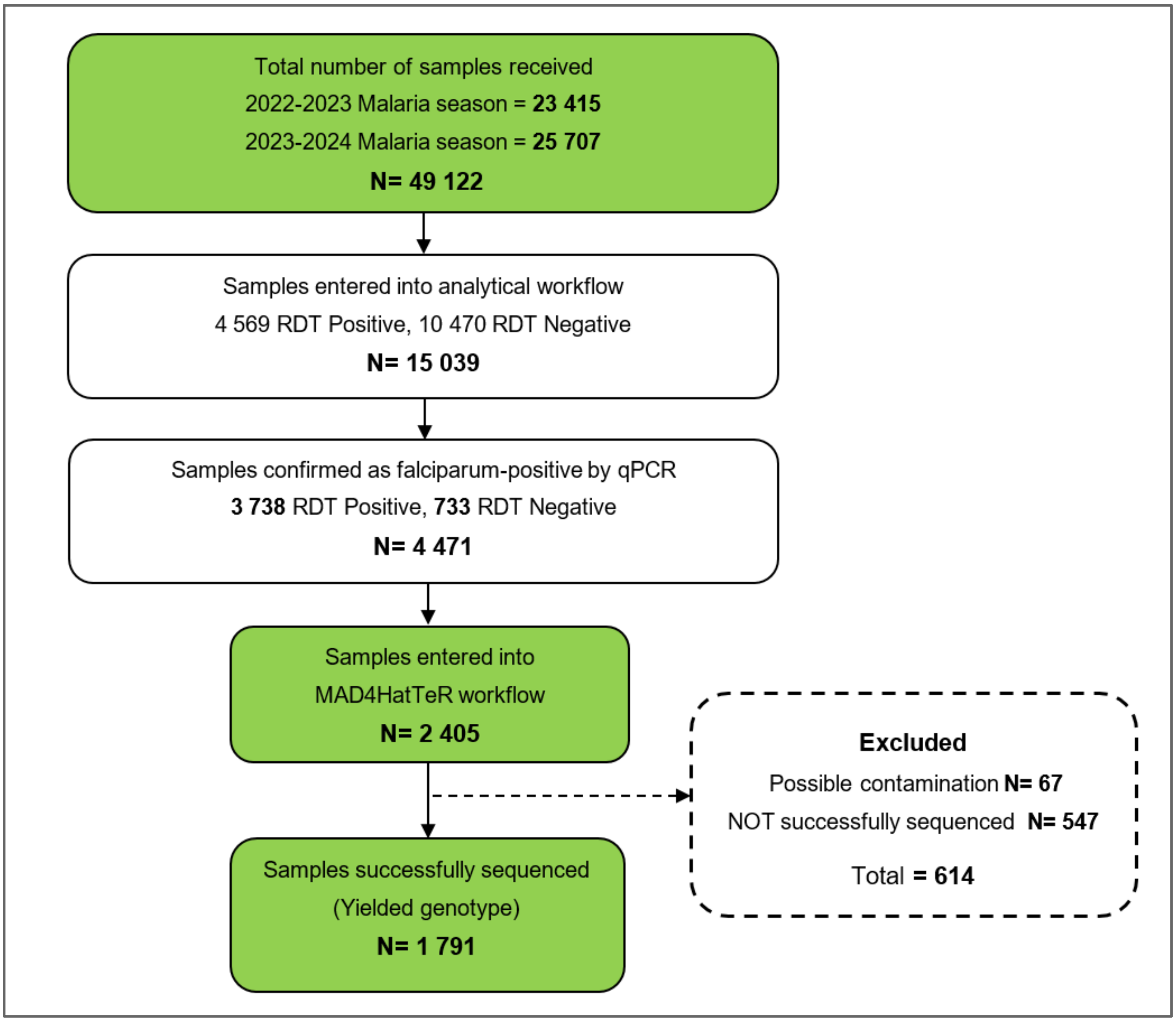

Figure S1: The number of samples received by the central laboratory at the National Institute for Communicable Diseases, Johannesburg, South Africa during the 2022/2023 and 2023/2024 malaria seasons, the number entered into analytical workflow, and the number successfully sequenced through the MAD^{4}HatTeR workflow.

Figure S2

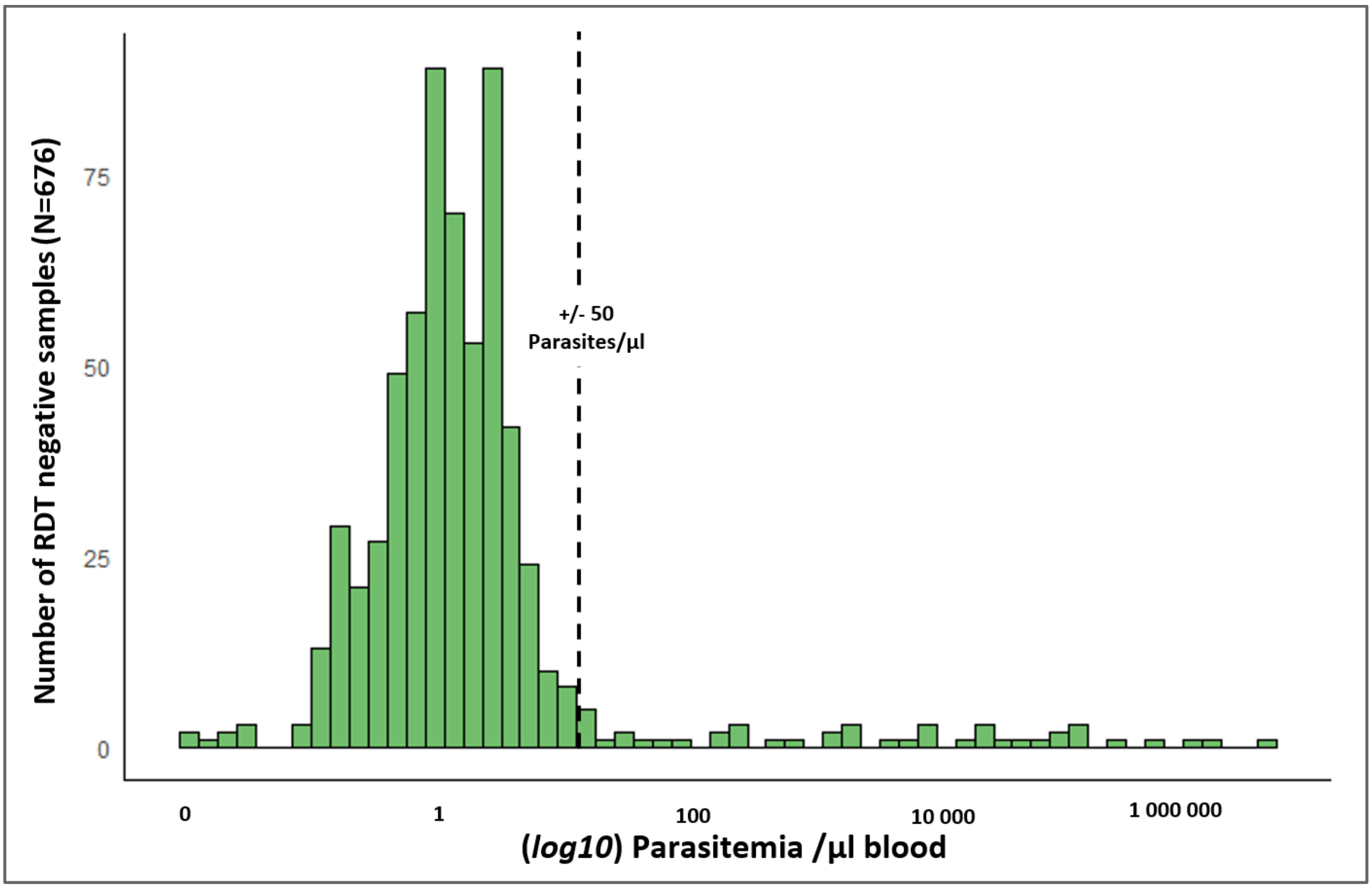

Figure S2: Parasitemia of RDT-negative but qPCR-positive samples detected over the study period

Figure S3

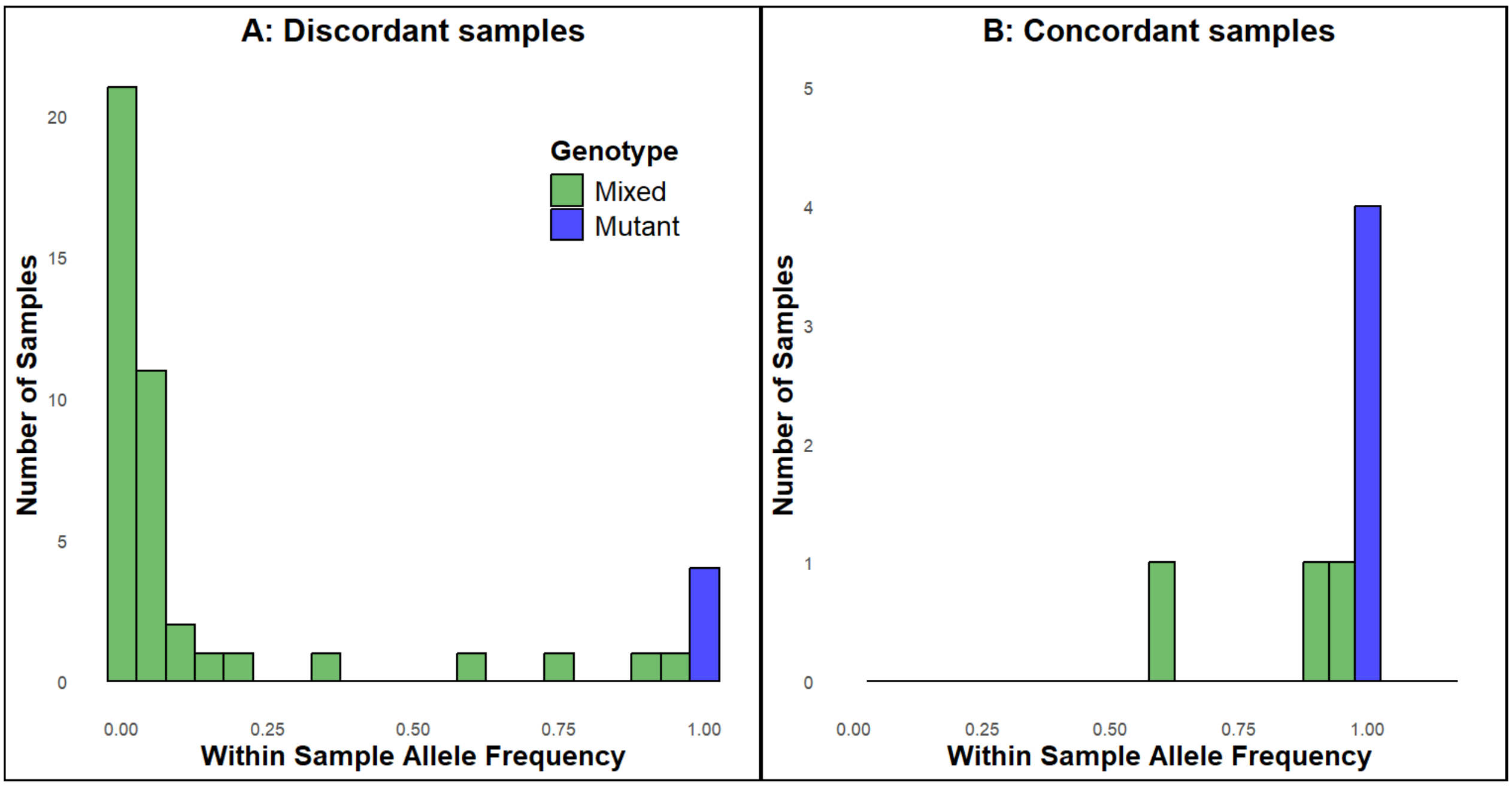

Figure S3: Within Sample Allele Frequencies (WSAF) for A) discordant and B) concordant samples containing a non-synonymous *kelch13* mutation
